# Endovascular repair of an abdominal aortic aneurysm with external iliac artery occlusion using VBX as the contralateral leg via axillary delivery: A case report

**DOI:** 10.1097/MD.0000000000037731

**Published:** 2024-04-05

**Authors:** Takafumi Akai, Takanori Kaneko, Takatoshi Furuya

**Affiliations:** a Department of Vascular Surgery, Asahi General Hospital, Chiba, Japan.

**Keywords:** AAA, EVAR, occluded external iliac artery

## Abstract

**Rationale:**

A hostile iliac access route is an important consideration when enforcing endovascular aneurysm repair (EVAR) for abdominal aortic aneurysms (AAA). Herein, we report a case of AAA with unilateral external iliac artery occlusion, for which bifurcated EVAR was successfully performed using a single femoral and brachial artery access.

**Patient concerns:**

A 76-year-old man who had undergone surgery for lung cancer 4.5 years prior was diagnosed AAA by computed tomography (CT).

**Diagnosis:**

Two and a half years before presentation, CT revealed an infrarenal 48 mm AAA, which had enlarged to 57 mm by 2 months preoperatively. CT identified occlusion from the right external iliac artery to the right common femoral artery, with no observed ischemic symptoms in his right leg. The right external iliac artery, occluded and atrophied, had a 1 to 2 mm diameter.

**Intervention:**

Surgery was commenced with the selection of a Zenith endovascular graft (Cook Medical) with an extended body length. Two Gore Viabahn VBX balloon expandable endoprosthesis (VBX; W.L. Gore & Associate) were delivered from the right axilla as the contralateral leg.

**Outcomes:**

CT scan on the 2nd day after surgery revealed no endoleaks.

**Lessons:**

While the long-term results remain uncertain, this method may serve as an option for EVAR in patients with unilateral external iliac artery occlusion.

## 1. Introduction

Hostile iliac access poses a challenge in enforcing endovascular aneurysm repair (EVAR), preventing EVAR in 6% to 15.4% of abdominal aortic aneurysm (AAA) patients.^[1]^ EVAR for AAA cases with occlusive iliac access typically involves endovascular revascularization, a surgical conduit,^[2–5]^ or the use of an aorto-uni-iliac (AUI) device.^[6]^ In cases where occlusion of the iliac artery extends to the common femoral artery (CFA), endarterectomy of the CFAs becomes necessary. Using an AUI device in cases with a patent common iliac artery (CIA) and occluded external iliac artery mandates embolism of the CIAs, necessitating consideration of revascularization, such as cross-over bypass. Herein, we describe a case of AAA with arterial occlusion from the external iliac artery (EIA) to the CFA following bifurcated EVAR using single femoral artery and brachial artery access.

## 2. Case report

The patient was a 76-year-old man who undergone surgery for lung cancer 4.5 years prior, resulting in pleural dissemination. Two and a half years before the present surgery, computed tomography (CT) revealed an infrarenal fusiform 48 mm AAA, which had enlarged to 57 mm, 2 months preoperatively. Additionally, he had a history of surgery for osteomyelitis of the right femur in elementary school and a fracture of the right lower limb due to a traffic accident in his 30s.

**Figure 1.**
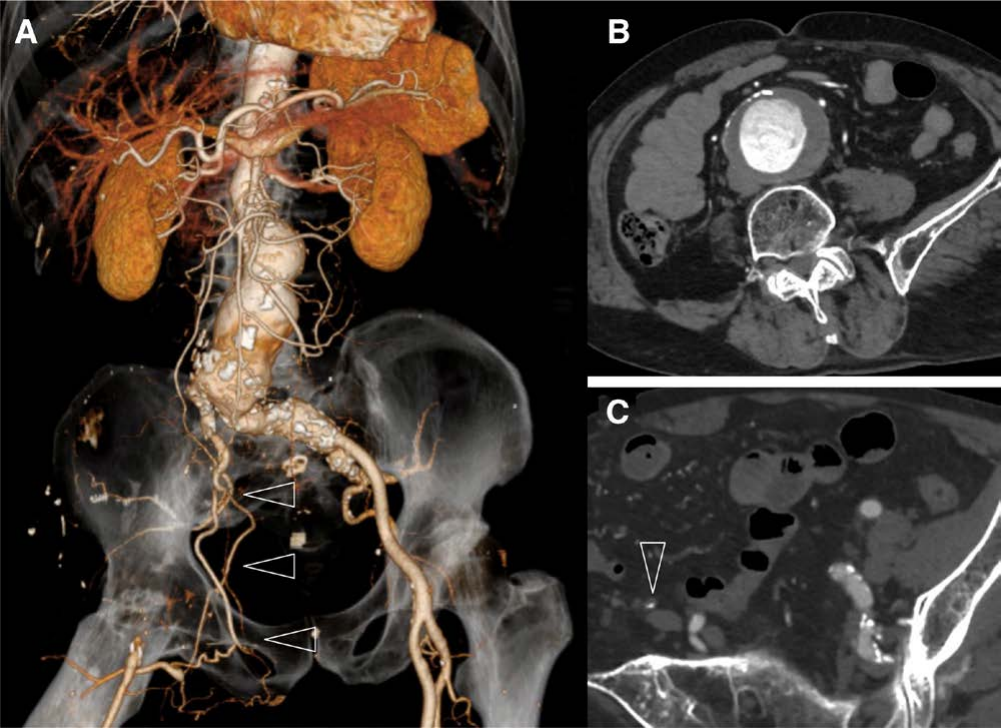
Preoperative enhanced CT images. (A) Volume rendering image of CT angiography showing occlusion of the right EIA to the CFA with collateral flow from the right IIA (arrow heads). (B) Axial view of CT shows AAA. (C) Contrast enhanced CT showing occlusion of the right EIA. The EIA was atrophied (arrowhead). AAA = abdominal aortic aneurysms, CFA = common femoral artery, CT = computed tomography, EIA = external iliac artery.

CT identified the AAA with a maximum minor axis of 57 mm and occlusion from the right EIA to the right CFA (Fig. 1, A and B). The right ankle brachial pressure index was 0.81, with no observed ischemic symptoms in his right leg. Given the stable lung cancer status and expected prognosis, we decided to perform EVAR.

The right EIA, which was occluded and atrophied, had a diameter of 1 to 2 mm (Fig. 1C). To maintain blood flow in the right lower extremity, EVAR was planned with landing on the right CIA, without right femoral access.

**Figure 2.**
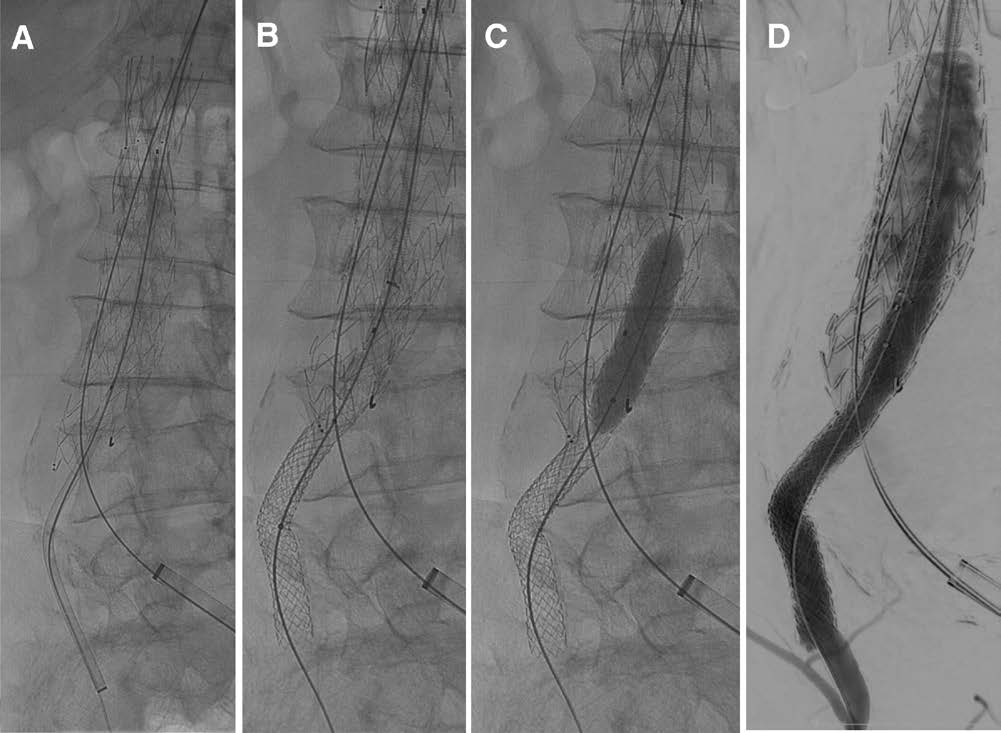
Intraoperative angiographic images. After placement of the main body, a guiding sheath was advanced from the right axilla to the right CIA (A), 2 VBXs were placed (B), and then crimped to the main body by post ballooning (C). Intraoperative angiography confirmed that there was no junction leak (D). CIA = common iliac artery.

**Figure 3.**
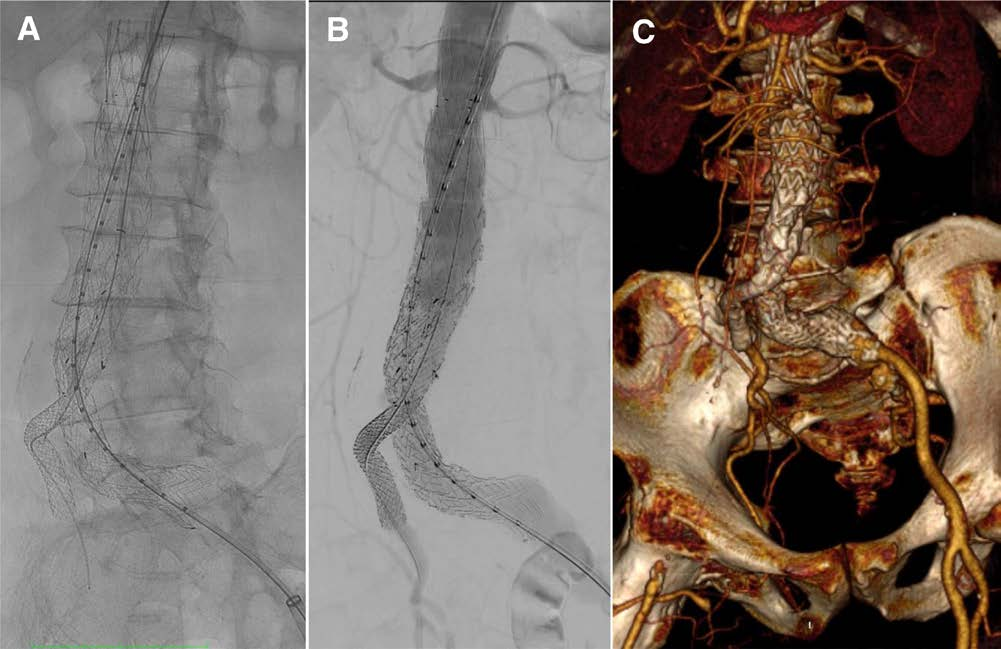
Angiographic image after stent graft placement (A). No endoleak was observed on the final intraoperative angiography (B), and postoperative CT (C). CT = computed tomography.

Under general anesthesia, surgery was commenced with the selection of a Zenith endovascular graft (Cook Medical) with an extended body length, placed below the renal artery. Due to stenosis of the left subclavian artery, the contralateral leg was delivered from the right axilla. The right CIA was selected using a wire from the right axilla, replaced with a stiff wire, and the guiding sheath was advanced into the right CIA (Fig. 2A). Because the normal EVAR leg could not reach the contralateral leg, we used Gore Viabahn VBX balloon expandable endoprosthesis (VBX; W.L. Gore & Associate). First, we placed a VBX of 7 × 78 mm in the CIA. There was a distance to the contralateral leg gate, and an additional VBX of 7 × 78 mm was subsequently placed and expanded to allow pressure contact with the main body (Fig. 2, B and C). The left CIA was dilated, a bell-bottom leg (Gore Excluder AAA [W. L. Gore & Associates] endoprosthesis leg) was placed on the ipsilateral leg, and kissing ballooning was performed. The final imaging showed no endoleaks (Fig. 3).

Postoperatively, the patient’s course was favorable. A CT scan on the 2nd day after surgery revealed no endoleaks. The right ankle brachial pressure index remained at 0.88, consistent with the preoperative value. The patient was discharged on postoperative day 8. However, the patient unfortunately died of exacerbated interstitial pneumonia 3 months after the operation.

## 3. Discussion

Severe neck cases pose significant challenges in EVAR, while poor access routes present additional obstacles. Solutions for cases of hostile access include the surgical conduit or internal endo-conduit method, or a more complicated approach.^[7]^ The internal endo-conduit method is often the first choice for iliac artery occlusion. In this case, the endo-conduit approach required a thromboendarterectomy of the right CFA. In addition, atrophy of the EIA was observed; therefore, it was unclear whether EVAR could be successfully performed using the internal endo-conduit method. While an AUI device is an effective alternative, our method is preferred as it involves embolization of the CIA, and allows for the possibility of a cross-over bypass.

In the present case, VBX was selected as the contralateral leg delivered from the axilla due to the inaccessibility of the normal EVAR leg. VBX, used as an internal iliac component for iliac branch devices,^[8,9]^ is believed to pose no problems with endoleaks or patency. Nabulsi et al^[10]^ reported a similar technique using the Viabhan (W. L. Gore & Associates) as the contralateral leg. Although long-term endoleaks and patency were not examined in this case, a self-expanding covered stent could be considered as an effective alternative for the contralateral leg.

The applicability of the proposed method to other cases depends on the successful guiding sheath and VBX delivery. If aortic bending hinders delivery, conversion to an AUI device may be necessary. Furthermore, upper extremity access itself may introduce complications,^[11]^ and it is necessary to carefully evaluate the risk before selecting this method.

In conclusion, herein, we present a case of AAA with EIA occlusion where the VBX was placed as the contralateral leg through axillary delivery. While the long-term results remain uncertain, this method may serve as an effective option for EVAR in patients with unilateral EIA occlusion.

## Author contributions

**Writing—original draft:** Takafumi Akai, Takanori Kaneko.

**Writing—review & editing:** Takafumi Akai.

**Conceptualization:** Takanori Kaneko, Takatoshi Furuya.

**Supervision:** Takanori Kaneko, Takatoshi Furuya.
